# 

**Published:** 1789

**Authors:** 


### LONDON

### MEDICAL JOURNAL.

VOLUME THE TENTH,

FOR THE YEAR 1789.

Surgeon Genl's Office LIBRARY
63449

LONDON:

PRINTED for the EDITOR,

And sold by JOSEPH JOHNSON, N°. 72,

ST. PAUL'S CHURCH YARD.

M,DCC,LXXXIX.

### THE

### LONDON

### MEDICAL JOURNAL,

FOR THE YEAR 1789,

PART THE FIRST.

### CATALOGUE OF BOOKS.

1. AN Eſſay on the Recovery of the apparently Dead. By *Charles Kite*, Member of the Corporation of Surgeons in London, and Surgeon at Graveſend in Kent: Being the Eſſay to which the Humane Society's Medal was adjudged. To which is prefixed Dr. *Lettſom*'s Addreſs on the Delivery of the Medal. 8vo. *Dilly*, London, 1788.

2. An Eſſay on the Rupture, called Hydrocele, explaining the Anatomy of the Parts affected; with Objections to the Inciſion, Seton, &c., in which is communicated an improved Method of radically curing that Diſorder with more Certainty and leſs Pain. By *Benjamin Humpage*, Surgeon, and Member of the Corporation of Surgeons in London. 8vo. *Reynell*, London, 1788.

3. The Works of *Thomas Sydenham*, M. D. on acute and chronic Diſeaſes; wherein their hiſtories and modes of cure, as recited by him, are delivered with accuracy and perſpicuity. To which are ſubjoined Notes, corrective and

 explanatory, explanatory, from the moſt eminent medical Writers; adapting the whole to the preſent improved ſtate of Phyſic, and ſhewing under what Claſſes, Order, and Genera, moſt of the complaints treated of are arranged by Noſologiſts: With a variety of Annotations. By *George Wallis*, M. D. 2 Vols. 8vo. *Robinſons*, London, 1788.

4. A Treatiſe on Diluents, and an Inquiry into the Diſeaſes of the Fluids of the Human Body, to aſcertain the Operation of Diluents upon them; with Dilution practically applied to particular Diſeaſes: wherein the Efficacy of Mineral Waters is conſidered. To which are prefixed Obſervations on common Water, ſo far as it reſpects the Subject of Attenuants. By *Thomas Jameſon*, Surgeon of His Majeſty's Navy. 8vo. *Murray*, London, 1788.

5. Obſervations on the Rupture of the Gravid Uterus; with the Sequel to Mrs. Manning's Caſe. By *Andrew Douglas*, M. D. Member of the College of Phyſicians, London; Conſulting Phyſician to the Lying-in Charity; and Phyſician to the Aſylum for Female Orphans. 8vo. *Johnſon*, London, 1789.

6. The Oeconomy of Health, or, A Medical Eſſay, containing new and familiar Inſtructions tions for the Attainment of Health, Happineſs, and Longevity; in which the Nature of the human Mind is accurately inveſtigated, and its Union and Connexion with the Body ſyſtematically explained. By *Andrew Harper*, late Surgeon to His Majeſty's Garriſon in the Bahama Iſlands. 8vo. *Stalker*, London, 1789.

7. A Treatiſe on the real Cauſe and Cure of Inſanity; in which the Nature and Diſtinctions of this Diſeaſe are fully explained, and the Treatment eſtabliſhed on new Principles. By *Andrew Harper*, Author of the Oeconomy of Health. 8vo. *Stalker*, London, 1789.

8. An Eſſay on the Fracture of the Patella, or Knee-pan; containing a new and efficacious Mode of treating that Accident, and obviating that Deformity and Lameneſs which ariſe from the old and common Mode of Treatment. Illuſtrated with Plates. To which are ſubjoined, Obſervations on the Fracture of the Olecranon. By *John Sheldon*, F. R. S. and Profeſſor of Anatomy in the Royal Academy of Arts, London. 8vo. *Johnſon*, London, 1789.

9. Diſſertatio Medica Inauguralis de Ictero. Auctore *Nicolao Bindon*, Hiberno. 8vo. Edinburgi, 1788.

10. Diſſertatio Medica Inauguralis de Dyſpepſia. Auctore *Gulielmo Jones Evans*, Hiberno. 8vo. Edin. 1788.

11. Diſſertatio Medica Inauguralis de Ophthalmia. Auctore *Joanne Eiſton*, Scoto. 8vo. Edin. 1788.

12. Diſſertatio Medica Inauguralis de Incubo. Auctore *Antonio Georgio Forbes*, ex inſulâ Sancti Chriſtophori. 8vo. Edin. 1788.

13. Diſſertatio Phyſica Inauguralis de Viſu. Auctore *Thoma Garnet*, Anglo. 8vo. Edin. 1788.

14. Diſſertatio Medica Inauguralis de Febribus; quaſdam ſuper iis Animadverſiones complectens. Auctore *Georgio Richardo Kittſon*, Hiberno. 8vo. Edin. 1788.

15. Tentamen Chemico-medicum Inaugurale de Calculo Urinario & Lithontripticis. Auctore *Georgio M'Farquhar Lawſon*, Indicus Occidentalis ex Inſula Jamaica. 8vo. Edin. 1788.

16. Diſſertatio Medica Inauguralis de Fluxus Menſtrui indole, cauſiſque. Auctore *Jacobo Chicheſter Maclaurin*, Londinenſi. 8vo. Edin. 1788.

17. Diſſertatio Medica Inauguralis de Alimento Hominum. Auctore *Joanne Ramſay*, Scoto. 8vo. Edin. 1788.

18. Diſ-

18. Differtatio Medica Inauguralis de Exercitatione. Auctore *Josepho Sherlock*, Hiberno. 8vo. Edin. 1788.

19. Differtatio Medica Inauguralis quædam de Ebrietate ejusque Effectibus in corpus Humanum complectens. Auctore *Thoma Trotter*, Scoto-Britanno. 8vo. Edin. 1788.

20. Differtatio Therapeutica quædam de Remediis Diureticis complectens. Auctore *Joanne Wilson*, Scoto-Britanno. 8vo. Edin. 1788.

21. Differtatio Medica Inauguralis de Dyfenteria contagiofa, præcipue de illa fpecie, quæ in Indiis Occidentalibus obfervatur. Auctore *James Barton*, Anglo. 8vo. Lugdun. Batav. 1788.

22. *Jufti Arnemann*, D. in Academia Georgia Augufta Profefforis Medicinæ publ. Commentatio de Aphthis, quæ ab ill. Reg. Societate Medic. Parifienfi 25 Aug. 1787, palmam alteram obtinuit. 8vo. Gottingæ, 1787.

23. Precis des Leçons publiques de Chimie & d'Hiftoire naturelle, qui fe font toutes les Années aux Ecoles de Medecine de l'Univerfité de Nanci; par M. *Nicolas*, Confeiller-Medecin du Roi, Profeffeur Royal de Chimie, Infpecteur honoraire des Mines de France, Membre de de l'Academie de la dite Ville, & de plusieurs autres. Seconde Edition, revué, corrigée, & augmentée. Tomes II. 8vo. Nanci, 1787.

24. Observations sur le Tetanos, ses Differences, ses Causes, ses Symptomes, avec le Traitement de cette Maladie, & les Moyens de la prevenir; precedées d'un Discours sur les Moyens de perfectionner la Medecine pratique, sous la Zone Torride, suivies d'Observations sur les Femmes Enceintes dans ces Regions, & sur la Conservation des nouveau-nés; avec un Rapprochement des Vices & des Abus des Hopitaux dans ces Pays. Par M. *Dazille*. 8vo. Paris, 1788.

25. Memoires sur les Fievres intermittentes malignes; par M. *Durand*, Docteur en Medecine de l'Université de Montpellier, Professeur du Cours public d'Accouchemens, etabli a Cahors, Correspondant de la Société Royale de Medecine. 8vo. Paris, 1788.

26. *R. Kirwan*'s Physisch-chemische Schriften, aus dem Englischen ueberfetzt von *Lor. Crell*, d. A. D. &c. 8vo. 3 Vols. Berlin, 1783-8.

27. Ueber die neuere Geschichte der Chirurgie in den K. K. Staaten; eine Rede, &c. *i. e.* On the modern History of Surgery in the Impe-

[illegible]

rial Dominions ; a Difcourfe by *J. Hunczoufky*. 4to. Vienna, 1787.

28. Obfervations fur un nouveau Moyen de guerir certaines Douleurs de Dents. Par M. *Pliffon*, Dentifte récu au College Royal de Chirurgie de la Ville de Lyon. 8vo. Lyon, 1788.

The cafes to which, according to this writer, his method is applicable, are thofe of caries attended with a collection of matter in the cavity of the tooth. His mode of treatment confifts in filing away the carious part of the tooth till he has laid open the whole of that part of the cavity correfponding with the caries. He mentions thirteen cafes in which this was done with fuccefs; and in all of thefe, we are told, a portion of fœtid matter was difcharged. He obferves, that, in cafes of this fort, filling up the cavity with gold leaf, or with lead, frequently ferves only to increafe the pain.

29. Ricerche fifiche fopra la Fermentazione vinofa, prefentate al Concorfo dell' Anno 1787 dal Padre *Gio. Batt.* da S. *Martino*, e qualificate con l'acceffit della reale Accademia de Georgofili di Firenze. 8vo. Florence, 1787.

30. Del Nitro Minerale, Memoria Storico-fifica dell Abb. *Alb. Fortis*. 8vo. 1787.

31. Esame della Teoria del Calor del celebre Inglese *Crawford*, con alcune nuove congetture sopra la Medesima Materia, di *Leopold. Vacca Berlinghieri.* 4to. Pisa, 1787.

32. Risposta del Dottor *Filippo de Carolis*, Ravennate, al Dottor *Ilario Andrea Piccioni*, da Monte dell' Olmo, intorno alla Questione: se sia o non sia contagiosa la Tisia polmonale. 8vo. Roma, 1788.

33. Opere Anatomiche e Cerusiche di *Ambrogio Bertrandi*, Professore di Chirurgia pratica nella R. Universita di Torino, Membro della reale Accademia di Chirurgia di Parigi, della Societa reale di Torino, e primo Chirurgo della S. R. M. del fu Re Carlo Emanuele, pubblicate, e accresciute di Note, e di Supplementi, dai Chirurghi *Gio. Antonio Penchienati* e *Gioanni Brugnone*, Professori nella Regia Universita, e Membri della reale Accademia delle Scienze di Torino. Tom. V. 8vo. Turin, 1786-7.

34. *J. F. Blumenbach*, Prof. Med. Gotting. Institutiones physiologicæ. 8vo. Gottingæ, 1787. c. Fig.

35. *J. F. Blumenbach* Specimen Physiologiæ comparatæ inter Animantia, calidi & frigidi sanguinis; præmissæ sunt de nisu formativo & de Generationis negotio, Observationes nuperæ,

Tabulis,

Tabulis æneis illustratæ. 4to. Gottingæ, 1787.

36. *G. F. Hoffmann* Vegetabilia Cryptogamica. Fasciculus primus cum Tabulis æneis VIII. 4to. Erlangæ, 1787.

37. *C. C. Krause*, Med. Doct. & Prof. Lipſ. Opuſcula Academica medico-practica, in hac Editione aucta & emendata, Edidit. *C. G. Kuhn.* Tom. I. 8vo. Lipſiæ, 1787.

38. *C. F. Reuſs*, Med. Doct. & Prof. Tubing. Diſpenſatorii univerſalis nuper editi Supplementum, continuationem ſiſtens remediorum medico-pharmaceuticorum; additamenta varia e Gmelin, Eſſig, Elwert, Lieblein & le Chandeliers ſcriptis chemico-pharmaceuticis, ut & e pharmacopœis Danica & Wurtembergica deprompta; morborum, & virium remediorum, conſpectum brevem alphabeticum, regulas denique medico-pharmaceuticas, & terminologiam. 8vo. Argentorat. 1787.

39. Nova Genera & Species Plantarum, ſeu Prodromus Deſcriptionum Vegetabilium, maximam partem incognitorum, quæ ſub itinere in Indiam Occidentalem Annis 1783-87 digeſſit *Olof Swartz*, M. D. 8vo. Stockholm, 1788.

40. *W. X. Janſen*, Phil. & Med. D. de Pelagra, Morbo in Mediolanenſi Ducatu endemio. 8vo. Lugd. Bat. 1788.

41. *Andreæ Jo. Georgii Murray*, Gottingenſis, Commentatio de Redintegratione partium Corporis animalis nexu ſuo ſolutarum vel amiſſarum; cui in concertatione Civium Academiæ Georgiæ Auguſtæ, iv Junii, 1787, locum a praemio ſecundum Ordo Medicorum adjudicavit. Cum Tabulis æneis. 4to. Gottingæ.

42. Diſputationes * Phyſicæ & Medicæ. Auctore *Michaele Franciſco Buniva*, Philoſophiæ & Med. Doctore, a Pinarolio. 8vo. Taurin. 1788.

43. Quædam circa Syſtematis abſorbentis Pathologiam. Auctore *Ludov. Formey*. 8vo. Halle, 1788.

44. *P. Chr. Fr. Werneri* Vermium inteſtinalium brevis Expoſitionis continuatio Tertia. Auct. *J. L. Fiſchero*. 8vo. Lipſiæ, 1788. c. Tab. æn. V.

45. De Fatis fauſtis & infauſtis Chirurgiæ, nec non ipſius interdum indiſſolubili Amicitia

* *Viz.* De generatione plantarum — De organis mulierum genitalibus — De hominum generatione — De generatione & propagatione vermium in canali cibario hoſpitantium, & morbis ab iiſdem originem habentibus — De anthelminticis.

cum cum Medicina coeterifque ftudiis liberalioribus ab ipfius origine ad noftra ufque tempora Commentatio hiftorica. Auctore *N. Riegels*. 8vo. Hafniæ, 1788.

46. Conftitutiones Epidemicæ per Cafulanum agrum graffatæ ab Anno 1787 ad Annum 1788. Auctore *Mauro Sartio*, Ruffienfi, ibidem publicis ftipendiis Medicinam faciente. 8vo. Faenza, 1788.

47. Theoretifch-practifche Beurtheilung des Scharbocks, oder art und weife, den Scharbock allein mit denjenigen mitteln, welche man wahrend der reife auf der fee befitft, zu heilen, im falle, dafs arzneyen fehlten, wie auch kurzer vortrag, wie man auf kauffartheyfchiffen, die weder arzt noch arzneyen mit fich fuhren, die leute gefund erhalten koenne, von *Dominicus Spedicati*, d. A. D. und Staabs-Chirurgus der Flotte. Aus der Ital. uebers. mit genehmhaltung der Ruff. Kaif. Medic. Collegii. *i. e.* A Theoretico-practical Difquifition on the Scurvy, or the Manner of curing that Difeafe, in cafes where there is a deficiency of Medicines, by the other means one is poffeffed of during Voyages at Sea; together with a fhort Effay on the Method of preferving the Health of the the Crew in Merchant Ships that take with them neither a Phyſician nor Medicines. By *Dominicus Spedicati*, M. D. Staff-Surgeon of the Fleet. Tranſlated from the Italian, with the approbation of the Imperial Ruſſian College of Phyſicians. 8vo. St. Peterſburg, 1787.

48. Geſchichte des Zinks in abſicht ſeines verhaltens gegen andere Koerper, und ſeiner anwendung auf Arzneywiſſenſchaft und Kunſte. *i. e.* Hiſtory of Zinc, with regard to its affinity with other Bodies, and its application to Phyſic and the Arts. By *G. Fr. Chriſtian Fuchs*. 8vo. Erfurt, 1788.

49. Ueber den Thieriſchen Magnetiſmus. *i. e.* On Animal Magnetiſm. By *C. Meiners*, Profeſſor of Moral Philoſophy at Gottingen. 8vo. Lemgo, 1788.

50. Bemerkungen und Beobachtungen ueber die Pocken in der Epidemie des Jahrs 1787. *i. e.* Remarks and Obſervations on the epidemical Small Pox of the Year 1787. By *G. F. Hildebrandt*, M. D. Profeſſor of Anatomy, &c. 8vo. Brunſwick, 1788.

51. Eſame Fiſico-chimico intorno alla Natura e Proprietà dell' Aria inflammabile paludoſa diretto a rintracciare i mezzi, co quali prevenire nire gli effetti perniciosi. Del Sig. *Moscheni.* 8vo. Lucca, 1788.

52. Dei Bagni di Montecatani trattato di *Alessandro Bicchierai,* Fiorentino. 4to. Firenze, 1788.

53. Raccolta dei Disègni delle Fabbriche Regie de Bagni di Montecatani nella Valdinievole. Folio. Firenze, 1788.

54. Atti della Reale Accademia delle Scienze, e Belle Lettere di Napoli dalla Fondazione sino all' Anno 1787. 4to. Napoli, 1788.

55. Essai sur le Phlogistique & sur la Constitution des Acides; traduit de l'Anglois de M. *Kirwan,* avec des Notes de MM. *de Morveau, Lavoisier, De la Place, Monge, Bertholet,* & *de Fourcroy.* 8vo. Paris, 1788.

56. Recherches, Memoires, & Observations, sur les Maladies Epizootiques de Saint Domingue, recueillis & publiés par le Cercle des Philadelphes du Cap François. 8vo. Paris, 1788.

57. Memoire qui a remporté le Prix, au Jugement de la Faculté de Medecine de Paris, le 22 Novembre, 1787, sur la Question proposée en ces Termes: — " Decrire la maladie

" du Mesentere, propre aux enfans, que l'on

" nomme vulgairement *Carreau*; l'envisager

" dans son principe; rechercher les causes qui

" la produisent, & exposer avec precision les

" moyens de la prevenir, & ceux de la guerir."

Par M. *Baumes*, Docteur en Medecine de la Faculté de Montpellier, &c. 4to. Nismes, 1788.

58. Manuel du Pharmacien, ou Instructions sur les differens Objets d'Etude necessaires aux Eleves en Pharmacie; par M. *de Machy*, Censeur Royal, & Demonstrateur d'Histoire naturelle au College de Pharmacie de Paris. 8vo. 2 Vols. Paris, 1788.

59. De l'Origine des Forces magnetiques; par M. *Prevost*, de l'Academie Royale des Sciences & Belles Lettres de Berlin, & Professeur Honoraire a l'Academie de Geneve. 8vo. Geneve, 1788.

